# *ATSAS 2.8*: a comprehensive data analysis suite for small-angle scattering from macromolecular solutions

**DOI:** 10.1107/S1600576717007786

**Published:** 2017-06-26

**Authors:** D. Franke, M. V. Petoukhov, P. V. Konarev, A. Panjkovich, A. Tuukkanen, H. D. T. Mertens, A. G. Kikhney, N. R. Hajizadeh, J. M. Franklin, C. M. Jeffries, D. I. Svergun

**Affiliations:** a European Molecular Biology Laboratory, Hamburg Outstation, Notkestrasse 85, D-22607 Hamburg, Germany; b Federal Scientific Research Centre ‘Crystallography and Photonics’ of Russian Academy of Sciences, Leninsky prospect 59, 119333 Moscow, Russian Federation; c A. N. Frumkin Institute of Physical Chemistry and Electrochemistry RAS, Leninsky prospect 31, 119071 Moscow, and N.N. Semenov Institute of Chemical Physics of Russian Academy of Sciences, Kosygina street 4, 119991 Moscow, Russian Federation; d National Research Centre ‘Kurchatov Institute’, ploshchad Kurchatova 1, 123182 Moscow, Russian Federation; e Department of Chemical Engineering, Stanford University, Stanford, California, USA

**Keywords:** small-angle scattering, data analysis, biological macromolecules, structural modelling, *ATSAS*

## Abstract

*ATSAS* is a comprehensive software suite for the analysis of small-angle scattering data from dilute solutions of biological macromolecules or nanoparticles. It contains applications for primary data processing and assessment, *ab initio* bead modelling, and model validation, as well as methods for the analysis of flexibility and mixtures. In addition, approaches are supported that utilize information from X-ray crystallography, nuclear magnetic resonance spectroscopy or atomistic homology modelling to construct hybrid models based on the scattering data. This article summarizes the progress made during the 2.5–2.8 *ATSAS* release series and highlights the latest developments. These include *AMBIMETER*, an assessment of the reconstruction ambiguity of experimental data; *DATCLASS*, a multiclass shape classification based on experimental data; *SASRES*, for estimating the resolution of *ab initio* model reconstructions; *CHROMIXS*, a convenient interface to analyse in-line size exclusion chromatography data;* SHANUM*, to evaluate the useful angular range in measured data; *SREFLEX*, to refine available high-resolution models using normal mode analysis; *SUPALM* for a rapid superposition of low- and high-resolution models; and *SASPy*, the *ATSAS* plugin for interactive modelling in *PyMOL*. All these features and other improvements are included in the *ATSAS* release 2.8, freely available for academic users from https://www.embl-hamburg.de/biosaxs/software.html.

## Abstract 2

Developments and improvements of the *ATSAS* software suite (versions 2.5–2.8) for analysis of small-angle scattering data of biological macromolecules or nanoparticles are described.

## 1. Introduction   

**Figure 1:**
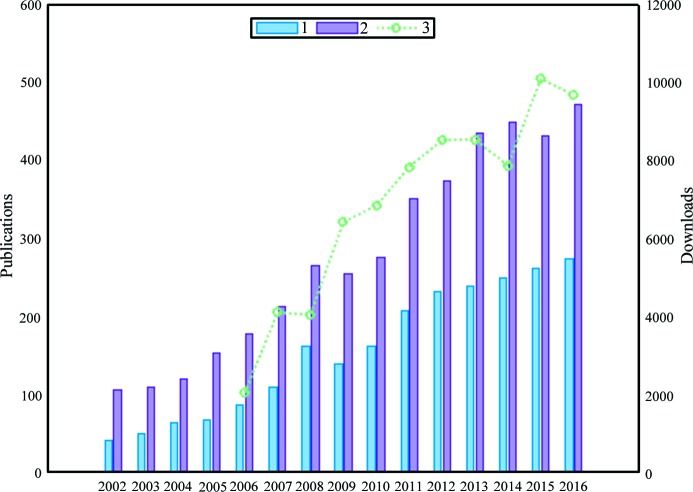
Publications referencing *ATSAS* (1) out of all publications using biological solution scattering (2; left axis). Download numbers of the *ATSAS* package since 2006 (3; right axis).

Small-angle scattering (SAS) is an increasingly popular technique for the structural characterization of macromolecular solutions, as shown by the constantly growing number of publications employing the method (Fig. 1 ▸). The application of both X-ray (SAXS) and neutron (SANS) scattering is diverse, ranging from the determination of low-resolution structures of individual macromolecules and higher-order complexes to probing flexibility, time-resolved conformational state(s) and structural changes in response to alterations in sample environment (Svergun *et al.*, 2013 ▸). The growing availability of advanced laboratory SAXS instruments, third-generation synchrotron X-ray sources, high-neutron-flux nuclear reactors and spallation sources enables researchers to access a wide range of experimental setups. In particular, recent developments include automated robotic sample changers for high-throughput studies, in-line analytical size exclusion chromatography coupled with SAXS (SEC-SAXS), microfluidic systems, shear and pressure cells, and components for sophisticated time-resolved experiments (Acerbo *et al.*, 2015 ▸; Blanchet *et al.*, 2015 ▸; Classen *et al.*, 2013 ▸; Graewert *et al.*, 2015 ▸; Jordan *et al.*, 2016 ▸; Kirby *et al.*, 2013 ▸; Li *et al.*, 2016 ▸; Pernot *et al.*, 2013 ▸).

In SAXS, one-dimensional scattering patterns are typically obtained by radially averaging the scattered photon counts measured on a two-dimensional detector around a beam stop that blocks the incident beam. The resulting one-dimensional scattering profiles are recorded as the scattering intensity *I*(*s*) as a function of the scattering vector *s*, where the momentum transfer *s* = 4πsinθ/λ, λ is the wavelength, and θ corresponds to half of the angle between incoming and scattered photons. Scattering of the background components, *e.g.* sample holder and solvent, has to be subtracted (Svergun *et al.*, 2013 ▸). In the most general case, the resulting intensities *I*(*s*) are on an arbitrary scale. It is to be noted that, because of the experimental setup, the forward scattering intensity at zero angle, *I*(0), has to be determined indirectly.

**Figure 2:**
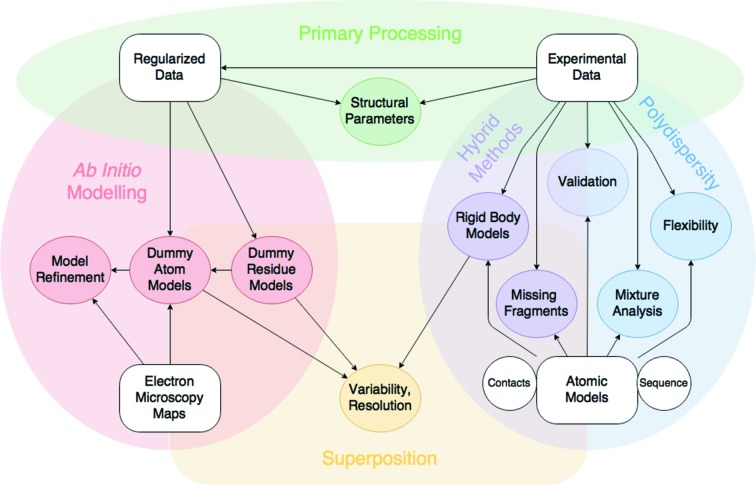
Overall scheme of the *ATSAS* application suite.

While advances in hardware development and automation are directly responsible for the kind of information researchers may collect during a measurement at any given beamline, the interpretation and modelling of data – which increasingly requires quality reporting and validation (Jacques *et al.*, 2012 ▸; Trewhella *et al.*, 2013 ▸) – is not trivial. To this end, the development of analytical and tools-based software to process and analyse SAS data is key for obtaining interpretable results and drawing clear conclusions from a scattering project (Fig. 2 ▸).

The SAS community is actively engaged in the development of data processing and analysis software packages. Some examples are *SASfit* (Breßler *et al.*, 2015 ▸), which fits the scattering data using a library of analytical expressions; *US-SOMO* (Brookes *et al.*, 2016 ▸), which integrates hydrodynamic parameters and SAS computation and fitting tools; *BioXTAS RAW* (Nielsen *et al.*, 2009 ▸) for raw data handling and SEC-SAXS data processing; *ScÅtter* (http://www.bioisis.net/tutorial/9), a Java-based application for SAXS data manipulation and analysis; and *Sasview* (http://www.sasview.org/), an open-source project by SANS facilities, providing data display, manipulation and fitting capabilities. The *ATSAS* suite of applications is a continuously developing cross-platform software package for the display, processing, analysis and modelling of solution SAXS and SANS data (Konarev *et al.*, 2006 ▸; Petoukhov *et al.*, 2007 ▸, 2012 ▸). Furthermore, *ATSAS* provides tools for hybrid approaches utilizing scattering data together with atomic structures from macromolecular crystallography (MX) and nuclear magnetic resonance (NMR), or shapes, envelopes or atomic structures from electron microscopy (EM). First released in 2003, the *ATSAS* package is arguably one of the most popular and comprehensive SAS data analysis and modelling platforms for isotropic SAXS and SANS on solutions of biological macromolecules and nanoparticles (Fig. 1 ▸). *ATSAS* programs cover the full spectrum of SAS data processing, manipulation and interactive tasks, ranging from radially averaging two-dimensional data into one-dimensional scattering curves, to the extraction of structural parameters, the computation of distance distribution functions and the reconstruction of three-dimensional models. *ATSAS* also offers tools to analyse polydisperse mixtures and flexible systems.

The most recent report presenting the overall and new features of *ATSAS 2.4* was published in 2012 (Petoukhov *et al.*, 2012 ▸). Here we describe the numerous additions developed across *ATSAS* versions 2.5 to 2.8, which have since become available to the scientific community, and provide a comprehensive overview of all of *ATSAS* with its multitude of applications to SAS data analysis.

## 2. Primary data processing   

Most data handling applications in the *ATSAS* suite have been designed and implemented with the same philosophy in mind: construct small and simple tools that interact well and may be used to build more complex analysis chains. This is most evident in the *DATTOOLS* suite, composed of modular command-line applications. Here the name *DATTOOLS* refers to a group of applications that generally operate on one or more experimental data file(s) and only carry out a single task, *e.g.* subtract two data sets, or estimate the radius of gyration (*R*_g_), the molecular weight (MW) or other structural parameters of the particle. As a convention, the names of the individual applications start with *DAT*, followed by the task performed, *e.g.* *DATOP* performs arithmetic operations on two input data sets and outputs the result. The functionality of the majority of *DATTOOLS* is most easily accessed through the interactive data analysis interface of *PRIMUS/qt*. In addition, with defined and standardized interfaces, the *DATTOOLS* also lend themselves to integration into automated data analysis pipelines (Brennich *et al.*, 2016 ▸; Franke *et al.*, 2012 ▸; Shkumatov & Strelkov, 2015 ▸) and graphical user interfaces (GUIs). The following briefly describes the *DATTOOLS* usage scenarios. Note that the automated analysis pipeline *SASFLOW* (Franke *et al.*, 2012 ▸), currently available at the BioSAXS beamline P12, EMBL, DESY, Hamburg, Germany (Blanchet *et al.*, 2015 ▸), and at the BL19U2 beamline, SSRF, Shanghai, China (Li *et al.*, 2016 ▸), is developed as part of the *ATSAS* suite, but is not routinely distributed to end-users. However, packages with *SASFLOW* and associated applications, *e.g.* *RADAVER* for radial averaging, are available on request.

All *DATTOOLS* use the open-source library *libsaxsdocument*, available as part of the open-source *saxsview* package (https://github.com/emblsaxs/saxsview), to read and write data. This library provides an abstraction for reading and writing data files in various formats, potentially including non-native *ATSAS* formats, for example the CanSAS XML format. External contributions to this open-source code supporting additional in- and output formats of other packages are encouraged.

*DATCMP* (assessment of one-dimensional data–data and data–model fits). During data collection and analysis investigators constantly face the problem of whether two or more data sets, experimental or calculated, may be considered similar up to random noise or whether significant differences exist. The reduced χ^2^ test has traditionally been employed to assess statistical discrepancies (Pearson, 1900 ▸). However, the validity of the reduced χ^2^ test requires an accurate assessment of the associated errors on the measured scattering intensities. Correctly specified error assessments may not be trivial, or indeed possible, to quantify. Therefore, alternatives have been sought (Trewhella *et al.*, 2013 ▸). The more recently developed CORMAP method (Franke *et al.*, 2015 ▸) does not require error estimates but provides the same statistical power to detect differences as a valid reduced χ^2^ test. The *DATCMP* utility in *ATSAS* implements the reduced χ^2^ test and CORMAP, as well as other tests, *e.g.* the Student t-test, for reference.

*DATAVER* and *DATOP* (arithmetic operations with intensities). If the compared one-dimensional scattering data, *e.g.* from two or more repeated measurements, are not considered significantly different according to *DATCMP*, they may be averaged by *DATAVER* to reduce the overall point-to-point variance of the experimental data. Basic arithmetic operations may also be performed using *DATOP*, such as the subtraction of background scattering contributions from unsubtracted one-dimensional profiles, or the addition or multiplication of/by a constant after averaging or normalization, for example, by concentration or sample transmission.

*DATMERGE*, *ALMERGE* and *DATADJUST* (intensity scaling and merging). Solution scattering data may be collected at several sample dilutions to evaluate the magnitude of interparticle effects. If it is assessed that the only difference between the background-subtracted profiles is the level of noise, then the data collected from the highest sample concentration may be used for further analysis. However, repulsive or attractive forces between the particles may lead to a notable change in the scattering intensities at very low angles. In such cases the low-angle data from a low-concentration sample, where the interparticle interaction effects may be negligible, may be merged with high-angle data from a higher concentration. Here, *DATMERGE* scales the manually selected ranges of two or more concentrations, such that the overlapping regions match and the merged data are provided on output. To simplify this process, *ALMERGE* may be used to find suitable overlap regions and to merge the data automatically. In addition, *ALMERGE* may extrapolate the data from multiple concentrations to an infinite dilution (Franke *et al.*, 2012 ▸). *DATADJUST* may be employed to match one data set to another by scaling and shifting, which may be useful for comparison of data collected under different conditions.

*DATABSOLUTE* (absolute scaling of intensities). The intensity values of the recorded SAXS data are by default arbitrary and therefore on a relative scale, but may be adjusted to an absolute scale [*I*(*s*), cm^−1^] by comparing the collected intensities with standards for which forward intensity can be calculated. Absolute calibration is most commonly performed using water (Orthaber *et al.*, 2000 ▸) or calibrated glassy carbon (Zhang *et al.*, 2010 ▸). *DATABSOLUTE* may be used, for example, to transform the collected data to an absolute scale using the scattering from water measured in the same environmental conditions as a sample, *e.g.* at the same temperature of the sample exposure unit, with identical exposure time and so on.

*SHANUM*, *DATCROP* and *DATREGRID* (angular grid manipulation). The angular range with meaningful signal-to-noise ratio may be determined by *SHANUM* and *DATSHANUM* (Konarev & Svergun, 2015 ▸). These applications employ Shannon representation of the scattering intensity (Shannon & Weaver, 1949 ▸), utilizing the fact that, for dilute monodisperse biological or nanoparticle solutions with a moderate degree of polydispersity, the particles in solution have a defined maximum dimension *D*_max_. Here, the useful angular range is defined, manually or automatically, *via* the number of effective Shannon channels that can be reliably determined from the data taking into account the oversampling and signal-to-noise ratio in the scattering intensities. The angular range of the data may be adjusted according to *DATSHANUM* by *DATCROP*, and more generally modified by *DATREGRID*. This includes re-binning the data to a template, joining data points to further reduce noise and the scaling of the grid, *e.g.* for conversion between inverse ångströms and inverse nanometres or to normalize by *R*_g_.

*DATRG* and *AUTORG* [*R*_g_ and *I*(0) calculated from the Guinier approximation]. Once data treatment is complete, for example after background subtraction and merging, *DATRG* may be used to compute the *R*_g_ of a particle and the forward scattering *I*(0) from a given input data range by means of the Guinier approximation (Guinier & Fournet, 1955 ▸). By default, the Guinier approximation for globular particles is assumed, but additional, modified Guinier approximations for rod-like and flat particles are also available, yielding the *R*_g_ of the particle cross section and thickness, respectively. In addition, as selecting the appropriate experimental data range for the Guinier approximation is often non-trivial, *AUTORG* (Petoukhov *et al.*, 2007 ▸) may be employed to identify the suitable Guinier interval automatically.

*DATGNOM* [automated calculation of real-space *p*(*r*) *versus r* distance distributions]. *DATGNOM*, previously known as *AUTOGNOM* (Petoukhov *et al.*, 2007 ▸), may be employed to automatically determine an approximation of the particle’s maximum dimension *D*_max_ that is used to calculate the inverse indirect Fourier transformation of the data and generate the probable real-space distance distribution, or *p*(*r*) profile. The program internally depends on *GNOM* (Svergun *et al.*, 1988 ▸; Svergun, 1992 ▸). In *ATSAS 2.6*, a modernized implementation of *GNOM*, then termed *GNOM5*, was introduced. Unlike previous versions, *GNOM5* may handle any number of simultaneously input data sets for a combined output, most useful for different detector positions in SANS experiments, and it uses all experimental data points without automatic rebinning. For Kratky cameras with slit smearing, experimental height and width beam profiles may be provided, as well as the parameterized slit definitions used by the original *GNOM*. The output file format has been modified to reflect these changes and is not directly compatible with the previous version. In *ATSAS 2.8*, the new *GNOM5* replaced the initial implementation of *GNOM* and is now accessible under this name.

*DATPOROD*, *DATMOW* and *DATVC* (geometry-based MW estimation). The *p*(*r*) profile, *i.e.* the output of *GNOM*/*DATGNOM*, may be used to estimate the Porod volume (*V*_p_) as well as concentration-independent estimates of the MW of proteins. The smoothed data are used by *DATPOROD* to estimate the excluded volume of an equivalent globular particle with uniform density. *DATPOROD*, like its predecessor *AUTOPOROD* (Petoukhov *et al.*, 2007 ▸), computes the volume by means of the Porod invariant (Porod, 1982 ▸). *DATMOW* and *DATVC* are implementations of the SAXS MoW MW estimation as originally described by Fischer *et al.* (2010 ▸) and the volume of correlation (*V*_c_) MW estimate by Rambo & Tainer (2013 ▸), respectively. These applications are included in the *ATSAS* suite for convenience: the MoW method has otherwise been described and implemented as an online service (http://www.if.sc.usp.br/~saxs/), and the *V*_c_ implementation is only available in the *ScÅtter* GUI. As with the other *DATTOOLS*, the provided binaries are designed to allow the inclusion of the respective methods in automated analysis scripts and other applications.

*DATABSMW* (MW estimation from absolute scale). Once the experimental data have been scaled to absolute scale (*DATABSOLUTE*) and normalized by concentration (*DATOP*), one may use *DATABSMW* to obtain the MW of the sample from the forward scattering intensity *I*(0) in combination with the partial specific volume and contrast. Various tools may be used to calculate the partial specific volume from the primary sequence, most easily using *SEQSTAT* [new in *ATSAS 2.8*, based on previously reported values (Harpaz *et al.*, 1994 ▸; Tsai *et al.*, 1999 ▸)], *MULCh* (Whitten *et al.*, 2008 ▸) or *NucProt* (Voss & Gerstein, 2005 ▸). In addition, the contrast may be calculated either through *CRYSOL* (Svergun *et al.*, 1995 ▸), if the atomic structure is available, or using the *Contrast* module in *MULCh*. Alternatively, empirically determined average values may be applied (Mylonas & Svergun, 2007 ▸) if none of the other methods are applicable or available.

## 3. Structural modelling using SAS data   

Beyond the computation of the structural parameters, SAS data are employed in various strategies to reconstruct low-resolution three-dimensional models. The approaches range from *ab initio* methods, which do not require any prior knowledge, to hybrid modelling where complementary structural models and additional biochemical and biophysical data are utilized. The general principle in all methods is the search for an optimal structural representation(s) of the studied system in such a way that the computed scattering of the models fits the experimental data. The target function to be optimized during modelling usually contains a measure of discrepancy between the experimental and calculated data and one or more penalty terms formulating additional requirements such as interconnectivity or distance constraints.

Recent additions to the *ATSAS* suite include *DATCLASS* and *AMBIMETER* for rapid shape classification and ambiguity assessment, respectively. Based on the predicted class and in conjunction with an ambiguity score, the most appropriate course of modelling may be chosen.

*DATCLASS* (shape classification of scattering profiles). The structural state, or states, of particles in solution is of significant interest prior to *ab initio* modelling. While compact particles may be more confidently modelled by bead-modelling applications, other shapes, for example flat particles, can become increasingly difficult to model without *a priori* information, for example symmetry (Volkov & Svergun, 2003 ▸), mainly owing to the inherent ambiguity of their scattering profiles (see *AMBIMETER*). In addition, flexible or intrinsically disordered systems often require quantitative ensemble methods (see *EOM*; §3.4) to describe their probable conformations as opposed to single-particle representations. The machine-learning-based multiclass classification utility *DATCLASS* provides an instant evaluation from the scattering data or calculated *p*(*r*) profile, to assess whether the particle is likely to be compact, extended or flat, and whether it might be hollow or better described as a random-like chain, as in the case of intrinsically disordered proteins (D. Franke, C. M. Jeffries & D. I. Svergun, manuscript in preparation).

*AMBIMETER* (evaluation of the shape ambiguity of a scattering profile). This tool rapidly quantifies the degree of ambiguity of an arbitrary scattering profile from a monodisperse solution of homogeneous particles and is based on a comprehensive library of scattering patterns from shape skeletons (up to seven closely packed beads) describing manifold low-resolution particle shape topologies (Petoukhov & Svergun, 2015 ▸). The corresponding scattering profiles are mapped to a normalized scale *I*/*I*(0) *versus sR*_g_ (Durand *et al.*, 2010 ▸), eliminating the size information and keeping the topology information only. The number of patterns similar to the given SAS experimental data provides a measure of the ambiguity associated with the data, and the logarithm of this value is reported as a quantitative *AMBIMETER* score. Generally, a score below 1.5 suggests that a unique *ab initio* shape restoration is possible, while a score above 2.5 points to potential ambiguity of the shapes restored from the given data set.

### 3.1. *Ab initio* modelling   

*Ab initio* shape analysis may be done on different levels, from comparison of scattering patterns with those of simple geometric shapes to dummy atom models (DAM) or, for proteins, dummy residue (DR) representations. Owing to the rotationally averaged nature of SAS data, the resulting *ab initio* models are intrinsically not unique (Volkov & Svergun, 2003 ▸). If *DATCLASS* indicates that a particle is likely to be flat, additional *a priori* information, *e.g.* symmetry constraints, should be provided to improve the reconstruction process. Furthermore, to evaluate the variability of the reconstruction, it is recommended to rerun the dummy atom and DR modelling applications (*DAMMIF*, *DAMMIN* or *GASBOR*) multiple times, 10–20 as a rule of thumb (possibly more if the *AMBIMETER* score is larger than 1.5). Different post-modelling options are available: selection of the most probable model of the whole set, clustering of models in the case of high ambiguity, model averaging and refinement, and, finally, evaluation of the model variability and resolution (Trewhella *et al.*, 2013 ▸).

*BODIES* (geometric shape evaluation). Following the traditional approach of visually comparing scattering patterns of geometrical objects, *BODIES* (Konarev *et al.*, 2006 ▸) varies the free parameters of simple geometric objects, *i.e*. the radius of a sphere, the sides of a parallelepiped, the length and diameter of a cylinder *etc.*, to fit the experimental data. Results may be obtained very quickly, but the fits beyond the Guinier region are generally poor.

*DAMMIN*, *DAMMIF* and *MONSA* (dummy atom modelling). Dummy atoms, or beads, are a collection of volume elements representing the molecular model. In this approach each bead represents an occupied volume element (generally, either particle phase or solvent phase), not an actual atom. After an initial random assignment of phases, *i.e*. whether a bead belongs to solvent or particle, the calculated scattering of the DAM is refined against the experimental scattering data by randomly switching the phases of beads while gradually reducing the probability of accepting those changes that do not improve the fit. Hereby the compactness and interconnectivity of the final model are ensured by application of penalty terms to the target function. *A priori* information about symmetry or anisometry may be taken into account as additional penalty terms. The described concept was initially implemented in *ATSAS* as the bead-modelling program *DAMMIN* (Svergun, 1999 ▸), and later in *DAMMIF* (Franke & Svergun, 2009 ▸). *DAMMIN* reconstructs a shape in a fixed search volume starting from a random approximation, while *DAMMIF* starts from a compact body as predicted by *DATCLASS* with useful modifications to search parameters, has an unlimited search volume and offers significant speed improvements. Besides the two single-phase bead-modelling programs, the *ATSAS* suite also includes *MONSA* (Svergun, 1999 ▸) for multiphase reconstructions. Here, multiple scattering patterns may be used as input to reconstruct multicomponent (*e.g.* protein–nucleic acid) complexes, most notably from X-ray and/or neutron contrast-variation data collections.

*DAM2DAM*, *EM2DAM*, *BODIES*, *DAMESV* (generating dummy atom models). Sometimes it may be convenient to obtain dummy atom models from different sources than bead modelling. For example, if high-resolution atomic models are converted to bead models, *DAM2DAM* may be employed to obtain a DAM approximation of the atomic structure. Density maps of electron microscopy may be converted by *EM2DAM*, which supports automatic threshold selection and, as of *ATSAS 2.8*, may apply constraints on the number of beads or graphs generated. Finally, *BODIES* may be employed to generate DAMs of the geometrical objects it supports. All DAMs generated by these applications may be used as a search volume for refinement without symmetry restrictions against SAXS data in *DAMMIN* or *MONSA*. If *a priori* information is available about multicomponent systems containing components with different average scattering length density, *DAMESV* may be used to generate starting DAMs with the appropriate volume fractions of each phase for subsequent *MONSA* refinement, for example against contrast-variation data.

*GASBOR* (DR modelling of proteins). The *ab initio* method implemented in the program *GASBOR* (Svergun *et al.*, 2001 ▸) utilizes a DR approach to model protein structures. Each amino acid is represented by a DR with an effective average scattering form factor centred at the approximate Cα position. Starting from a random configuration, the program performs a Monte Carlo-based search of the spatial distribution of DRs inside a spherical search volume with diameter *D*_max_ equal to the maximum particle size. The resulting DR assembly is required to be compatible with a typical distribution of backbone Cα atoms in proteins and the scattering computed from this assembly should fit the experimental data. This approach requires *a priori* knowledge about the number of amino acids (typically known from the protein sequence) and offers a somewhat more detailed description of the low-resolution structure as compared to uniform-density bead modelling.

*ALPRAXIN* (alignment to principal axes). The module is useful for representation purposes to align the principal axes of a model to the coordinate axes. However, for an accurate and complete superposition of models this is generally insufficient.

*SUPCOMB* and *SUPALM* (superposition of low- and high-resolution models). The basic requirement for any *ab initio* model analysis is the availability of a suitable approach to spatially align and compare models. This includes the alignment of individual models of a generated ensemble, and the alignment of *ab initio* bead models with atomic models or, in some cases, with electron density maps obtained from EM. As the number of dummy beads is not fixed in *ab initio* shape reconstruction and as the number of dummy beads does not usually coincide with the number of actual atoms of high-resolution structures, a simple one-on-one overlap algorithm such as, for example, the Kabsch algorithm (Kabsch, 1976 ▸) would not be suitable to align dummy atom models by themselves, or dummy atom models and high-resolution crystal structures. For the absence of one-to-one correspondence, the normalized spatial discrepancy (NSD) was developed (Kozin & Svergun, 2001 ▸). *SUPCOMB* superimposes two models in real space, be they low-resolution shapes or high-resolution crystal structures, by minimizing the NSD between them; for ideally superimposed similar objects, the NSD tends towards 0.0, and it exceeds 1.0 if the objects substantially differ from one another. Note that the magnitude of the NSD may also depend on the type of representation, for example if all atoms or only Cα are used, whether there are dummy beads involved and so on. So the conclusions on non-similarity should be taken with caution. A more recent addition to *ATSAS*, *SUPALM* (Konarev, Petoukhov & Svergun, 2016 ▸), overlaps models in reciprocal space. *SUPALM* is based on the spherical harmonics representation of the scattering amplitudes and uses a normalized integrated cross-term of the scattering amplitudes as calculated by *CRYSOL* (Svergun *et al.*, 1995 ▸) as a proximity measure between model representations. While *SUPALM* provides similar overlap results to *SUPCOMB*, it is about ten times faster for large macromolecules. In addition, *SUPALM* is especially useful for the alignment of inhomogeneous particles having components with different scattering length density, for example protein–RNA or protein–DNA complexes. Both alignment procedures also allow the comparison of bead models with electron microscopy density maps converted into bead models *via* *EM2DAM* and with atomic resolution structural models.

*DAMSUP*, *DAMSEL* and *DAMCLUST* (automated superposition and clustering of models). Repeated runs of *ab initio* modelling applications are necessary for robust reconstruction. However, the final/best model must be selected from the often large group of models generated. To make this task manageable, one may use *DAMSUP* to pairwise superimpose all models by *SUPCOMB* to compute the respective NSD and the overall resolution (see *SASRES* below). *DAMSEL* removes spatial outliers and selects the largest group of similar models within any given cohort. In addition, *DAMSEL* will select the most probable model as the one having the smallest average NSD relative to the other selected models. In contrast, *DAMCLUST* groups the selected models into clusters of similarity, which is especially interesting for those SAS datasets with high *AMBIMETER* ambiguity scores.

*DAMAVER* (averaging models). Any group of models, for example those selected by *DAMSEL* or the grouped clusters of *DAMCLUST*, may be averaged by *DAMAVER* (Volkov & Svergun, 2003 ▸), resulting in a model occupancy envelope. Note that the scattering of this envelope does not fit the experimental data and it is not recommended to use these models for publications; the most probable or refined model (see below) should be presented instead. However, the envelope may be employed as a search volume for further shape refinement (see below). Also note that, while it is possible to treat DR models of *GASBOR* in this manner, the DRs would be broken into dummy beads and information may be lost.

*DAMFILT* (filtering out low-occupancy beads in averaged models). *DAMFILT* generates a more compact representation of the occupancy envelope by removing low-occupancy or loosely connected beads (Volkov & Svergun, 2003 ▸). The envelope has a volume equal to the average *DAMMIN*/*F* reconstruction, but still, the scattering computed from this filtered envelope does generally not fit the experimental data and should not be included in publications.

*DAMSTART* (post-refinement of models after averaging). On the basis of the occupancy envelope generated by *DAMAVER*, *DAMSTART* creates a search volume with a fixed model core and a limited solvent shell for the refinement of the dummy atom arrangement with *DAMMIN*. The refined solution should fit the data and is recommended for publication of *ab initio* modelling results.

**Figure 3:**
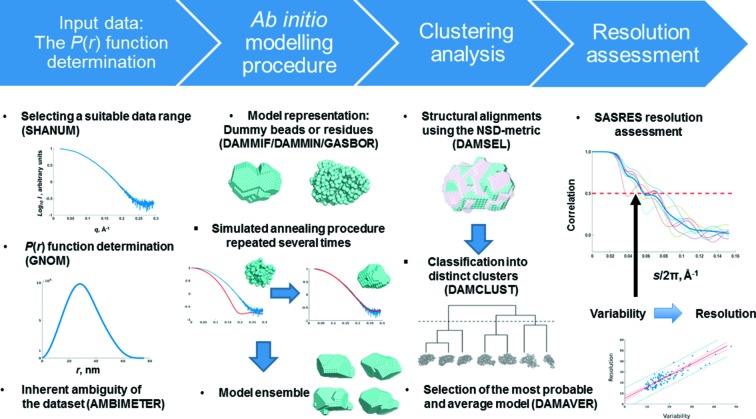
Overview of SAS-based *ab initio* modelling. The modelling process starts by selection of a suitable data range and determination of the corresponding *p*(*r*) function. The chosen *ab initio* modelling approach is repeated several times in order to explore the available solution space, and the generated models are grouped according to their structural similarity. The most probable and average models are selected on the basis of the clustering. Pairwise FSC functions of the structurally aligned bead or dummy residue models are computed. The average of all pairwise FSC functions is used to determine the variability estimate Δ_ens_ as 2π/*s*_ens_, where *s*_ens_ is the momentum transfer value at which the average FSC drops below 0.5. The corresponding resolution is estimated from the variability.

*SASRES* (quality assessment and resolution). Ambiguity assessments and averaging procedures provide useful qualitative estimates of the relative spatial distribution of models within an ensemble, but they do not yield an assessment of the model resolution. Deducing the resolution from the maximum momentum transfer value *s*_max_ used to build a SAS model only provides the nominal theoretical limit of 2π/*s*_max_, which is of little practical meaning because of the inherent ambiguity of SAS data. As *ab initio* models do not contain information at the level of atomic detail, validation-based approaches utilized in other structural biology methods are not easily applicable and, until recently, no methods to assess resolution were available. Instead, the resolution of *ab initio* SAS models can be deduced from their variability using the Fourier shell correlation (FSC) approach. This approach is widely used in EM (Penczek, 2010 ▸), where the FSC is computed between the scattering amplitudes of the reconstructions based on independent experimental sets. FSC decreases with increasing momentum transfer, reflecting the loss of structural similarity; typically a threshold of FSC dropped to 0.5 is taken to assess the resolution. In SAS, there is only one data set, but multiple *ab initio* models can be constructed that fit the data. It was demonstrated (Tuukkanen *et al.*, 2016 ▸) that the average FSC function over an ensemble of pairwise-aligned *ab initio* models (reflecting their variability) can be linked also to the resolution of the shape reconstruction. The program *SASRES* implements the FSC approach using fast spherical harmonics computations in reciprocal space (Fig. 3 ▸). The resolution estimate is automatically done in *ATSAS 2.8* as a part of the *DAMAVER* averaging procedures, specifically by *DAMSEL*, and is recorded in the corresponding *DAMSEL* log file. The FSC-based resolution analysis provides a quality measure according to the requirements of the wwPDB Small Angle Scattering Task Force (Trewhella *et al.*, 2013 ▸) and should be reported in publications and depositions of SAS data and models.

### 3.2. Hybrid modelling methods   

Multiple techniques in structural biology may be combined with hybrid modelling approaches. Hybrid methods utilize scattering data together with atomic structures from MX and NMR, or shapes and envelopes from EM. Such a combination may yield results not accessible to any one technique.

*DAM2IS* (scattering patterns of dummy atom models). The program *DAM2IS* calculates the scattering profiles of dummy atom models generated by modelling applications like *DAMMIN*, *DAMMIF* and *MONSA* and from transformational operations like *DAM2DAM* and *EM2DAM*.

*CRYSOL*, *CRYSON* and *CRYSOL 3* (scattering patterns of high-resolution models). The applications *CRYSOL* (Svergun *et al.*, 1995 ▸) for X-rays and *CRYSON* (Svergun *et al.*, 1998 ▸) for neutrons are used to calculate model scattering profiles from high-resolution atomic structures, with options to evaluate the fit of the model profile to experimental data. The discrepancy of data-model fits is reported using the reduced χ^2^ test. *CRYSOL* and *CRYSON* have options to adjust the X-ray or neutron contrast and take into account the contrast of the hydration shell surrounding macromolecules. In addition, the excess scattering density of the hydration shell, the average atomic group radius and the related total excluded volume may be adjusted. *CRYSOL* and *CRYSON* are particularly useful when comparing the atomic structures of macromolecules obtained from crystallography with the scattering patterns measured from the same macromolecules in solution, for example to identify the biologically relevant oligomeric states or to discriminate between alternative conformations. The original implementation of *CRYSOL* represents the hydration shell by an envelope function. As the accuracy of this approach may be limited for complex shapes, *ATSAS 2.8* also contains a preliminary version of *CRYSOL 3*, which represents the hydration shell as dummy beads covering the particle surface. These beads are then divided into three classes: (*a*) internal water in cavities, (*b*) water shell on the outer convex surface and (*c*) water on the concave surface. The mobility and thus the contrast of the beads may vary depending on the location. This more sophisticated water-beads handling permits one to achieve a better prediction of the scattering at higher angles, and the test version of *CRYSOL 3* is therefore made available (manuscript in preparation).

**Figure 4:**
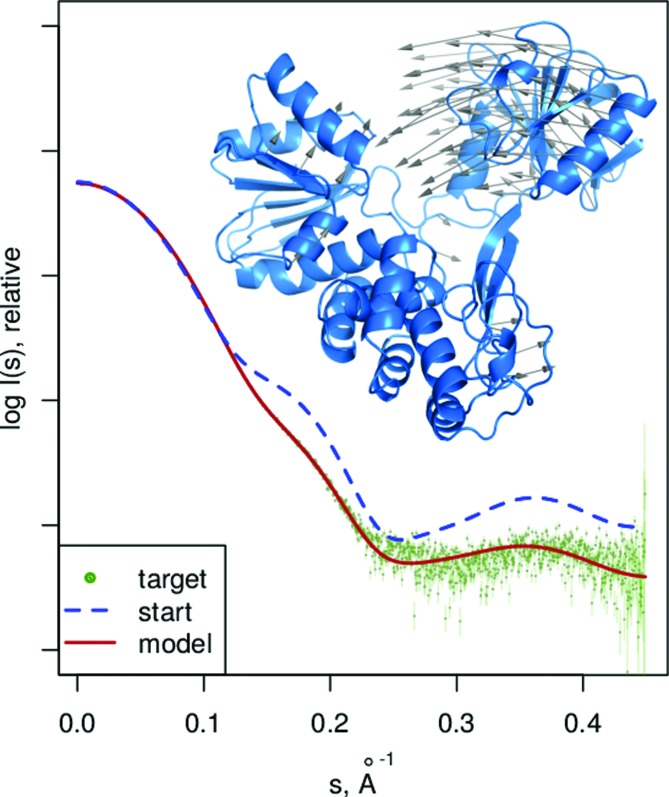
*SREFLEX*, flexible refinement of high-resolution models based on SAXS and normal mode analysis. Vectors show the conformational change modelled for hepatitis C virus NS3 helicase by *SREFLEX* when starting from PDB code 8ohm (Cho *et al.*, 1998 ▸) (unbound conformation, blue) guided by the SAXS profile of a nucleotide-bound conformation (dots, simulated from PDB code 3kqn; Gu & Rice, 2010 ▸).

*SREFLEX* (normal mode refinement of atomistic models from MX). Owing to the static nature of the conformational snapshots provided by MX and the typically less physiological conditions of the crystallization process, MX structures often provide a biased sampling of the conformational space explored by the macromolecule in solution (Fig. 4 ▸). *SREFLEX* (Panjkovich & Svergun, 2016*b* ▸) implements a hybrid modelling approach using normal mode analysis (NMA) (Delarue & Sanejouand, 2002 ▸) to explore the conformational space of high-resolution models of biological macromolecules to fit the available SAXS data. Using the principles of NMA, *SREFLEX* divides a structure into subdomains that are treated as rigid bodies during the first stages of refinement. The partitioning allows the application to probe large conformational changes while limiting the stereochemical distortion of the structure. In the subsequent second stage of refinement subdomain restraints are discarded, allowing the residues to move more independently. In both stages, normal modes are explored in a hierarchical manner, starting with low-frequency modes that correspond to large global rearrangements and continuing with higher-frequency modes to refine smaller and more localized movements. *SREFLEX* is useful for a ‘soft’ refinement of the high-resolution models of biological macromolecules which, taken as such, provide moderate misfits to the experimental SAXS data.

*SASREF* and *SASREFCV* (rigid-body modelling of monodisperse systems). In studies of large macromolecular complexes it is quite common that the structures of individual subunits are known from crystallography or NMR, but not the structure of the entire assembly. In this situation, solution scattering can be used for rigid-body modelling. The program *SASREF* (Petoukhov & Svergun, 2005 ▸) starts with random positions and orientations of the subunits with known atomic structures and employs simulated annealing to move and rotate them in order to construct an interconnected assembly without steric clashes, which fits the experimental SAXS data. The program *SASREFCV* is similar to *SASREF* except that it is specifically tailored to refine the structures of macromolecular complexes against multiple contrast-variation or contrast-matching datasets as performed using SANS. The program allows for the input of D_2_O content in the solvent as well as perdeuteration levels of individual subunits. These values are then utilized for the assignment of the contrast for each subunit in the given dataset. *SASREFCV* also has options for modelling complexes using both SAXS and SANS contrast-variation data.

*BUNCH* and *CORAL* (addition of missing fragments to proteins and their complexes). The above *SASREF* approach for SAXS may also be applied to studies of multidomain proteins, whereby the individual domains play the role of rigid bodies. Modular proteins may contain inter-domain linkers where the high-resolution atomic structures are not known, for example because of conformational heterogeneity, or may change conformation in solution. The hybrid modelling program *BUNCH* (Petoukhov & Svergun, 2005 ▸) represents the protein as a consecutive string of high-resolution atomic models of domains connected by DR chains that serve as inter-domain linkers. The program employs simulated annealing to search for the optimal arrangement of the domains and the possible conformations of the DR linkers connecting them. The SAXS data from the full-length protein may be simultaneously fitted with the scattering profiles from deletion mutants, if available. Whereas *BUNCH* is typically employed to model single-polypeptide chains and their symmetry-related oligomers, *CORAL* (Petoukhov *et al.*, 2012 ▸) is used to model missing fragments and linkers of multi-subunit complexes and assemblies that includes protein–protein and protein–polynucleotide systems. Unlike *BUNCH*, the DR linkers are not generated on the fly but are taken from a pre-generated library of linker conformations that serve as placeholders for the missing portions of the structure. In the modelling procedure, rigid-body rearrangements are found which are consistent with an insertion of a random DR linker of the appropriate length stored in the library.

*GLYCOSYLATION* (addition of carbohydrates to glycoproteins). The molecular structures of glycoproteins available from the Protein Data Bank (PDB) often contain the atomic coordinates of the protein part but the coordinates of the carbohydrate atoms are missing owing to flexibility and/or uncertainty of their position. The scattering from N- or O-linked carbohydrates may contribute significantly to the total scattering of a glycoprotein, and it is necessary to properly account for this term in SAS data analysis. We have developed the *GLYCOSYLATION* tool for the *ad hoc* addition of carbohydrates to protein models, which utilizes a lookup table of 50 pre-computed glycan structures in the range of mol­ecular weights from 319 to 6800 Da. The structures were generated using the server *Sweet2* (Bohne *et al.*, 1998 ▸, 1999 ▸), which models the three-dimensional structure of saccharides from their sequences using standard nomenclature on a protein surface. *GLYCOSYLATION* can run in automatic mode, where randomly selected glycan groups from the database are attached in random orientations to randomly selected asparagine (ASN) or serine (SER) residues found on the surface of the given protein structure. Alternatively, the user may specify the exact glycosylation used for desired ASN and SER residues. The use of the subunit models with amended sugars may significantly improve the accuracy of intensity calculation in the course of rigid-body modelling compared to the case when the protein models are used ‘as is’ without taking into account the glycosylation.

### 3.3. Polydisperse systems   

The requirement of monodispersity is typically a prerequisite for reliable three-dimensional reconstructions from SAXS data. In some systems like weak oligomers and transient complexes, highly flexible systems, or nanoparticles, polydispersity may be an inherent property of the sample. Therefore, the measured scattering intensity will be a linear combination of the scattering of each component species weighted by the product of their respective volume fraction and contrast squared, assuming that there is no interaction between the particles. Listed below are the options available in *ATSAS* for mixtures of different types of particles. Note that for systems with similar particle shapes and continuous size distributions, for example synthetic nanoparticles, one may utilize an option in *GNOM* to compute the size distribution function while typically assuming a spherical particle shape.

*MIXTURE* (multicomponent system of geometrical objects). This is a generalized version of *BODIES* to analyse multicomponent and potentially interacting systems in terms of mixtures of simple geometric objects (Konarev *et al.*, 2003 ▸). *MIXTURE* fits an ensemble of up to ten geometric objects (allowed are core–shell spheres, core–shell cylinders, ellipsoids and dumbbells) to an experimental dataset while taking the size distributions and the structure factors of the interparticle interactions into account.

*OLIGOMER*, *FFMAKER* and *SVDPLOT* (determination of volume fractions of components in mixtures). The mixture analysis application *OLIGOMER* calculates volume fractions of the individual components in a mixture when the scattering patterns or high-resolution structures of the components are available. Its support application *FFMAKER* combines the form factors of any given number of components calculated from their atomic structures into a conglomerated form-factor input file for *OLIGOMER*. *OLIGOMER* then minimizes the discrepancy between the calculated composite curve from the mixture and the experimental data *via* optimizing the (non-negative) volume fraction contributions of the individual component form factors. Alternatively, estimates of the number of components present in a sample may be obtained from *SVDPLOT* (Konarev *et al.*, 2003 ▸) by means of singular value decomposition (SVD), for example to identify concentration-dependent structural transitions in monomer–dimer and multicomponent equilibrium mixtures or to evaluate the effects of changing the sample environment or the impact of mutations on global structural states. The SVD approach may be particularly useful for tracking the evolution of structural changes through time, *i.e.* time-resolved SAXS/SANS studies. Additional constraints may be manually specified in *SVDPLOT* that limit the volume or number fractions of the components in instances where the molar ratios between the individual components have been determined using alternative methods.

*GASBORMX* (*ab initio* modelling of mixtures). If a protein exists in solution as an equilibrium between a monomer and a higher-order symmetry oligomer, for example monomer–dimers, monomer–hexamers *etc.*, the *ab initio* modelling program *GASBORMX* (Petoukhov *et al.*, 2012 ▸, 2013 ▸) may be used to simultaneously model both the monomer and oligomer structures against the SAXS data. The program generates DR models of a symmetric homo-oligomer while taking into account the volume fraction of dissociated monomers. Given the fact that the monomer simply represents the asymmetric part of the intact oligomer, there is just one additional fitting parameter as compared to the monodisperse scenario, namely the volume fraction of the oligomeric particles in the mixture.

*SASREFMX* (rigid-body modelling of mixtures). For the case where the high-resolution structures of the components of a multimeric complex are available, rigid-body modelling of the entire assembly in combination with disassociation products can be performed by *SASREFMX* (Petoukhov *et al.*, 2012 ▸). While *SASREF* assumes 100% association of the components, with *SASREFMX* it becomes possible to model complexes and oligomers with lower binding affinities, for example through the analysis of concentration series data. Under the assumption that the individual components do not undergo significant conformational changes on forming a complex, the experimental data are fitted by a linear combination of the scattering computed from the dissociation products and that from the entire complex. Here, the volume fractions of dissociation products in the mixture are included as additional optimization parameters of the fit.

### 3.4. Flexible and unfolded proteins   

Flexibility is a common feature of biological macromolecules that often drives function. Structural disorder in multidomain and intrinsically disordered proteins is observed in 40% of proteins encoded in the human genome (Chouard, 2011 ▸). By their very nature proteins with significantly disordered and flexible regions prove to be difficult targets for high-resolution structure determination and are in most cases impossible to crystallize. SAXS provides one of the best methods for studying the structure and degree of disorder of these challenging systems.

**Figure 5:**
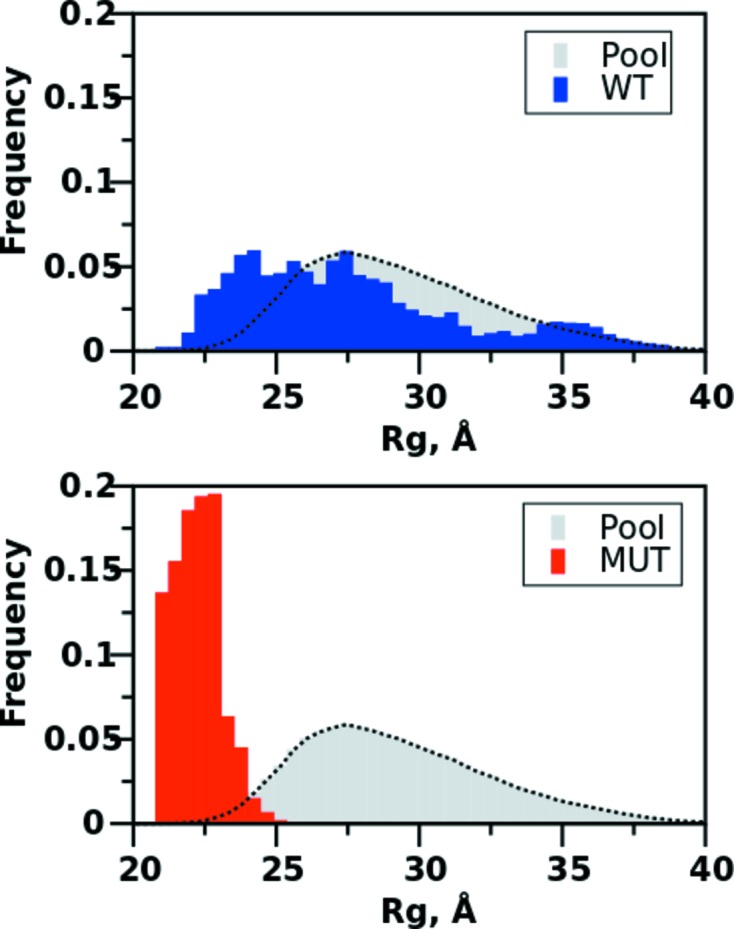
Ensemble optimization method (*EOM*). *R*_g_ parameter distributions for wild type (WT, upper panel) and a disulphide-stabilized mutant (MUT, lower panel) of urokinase plasminogen activator protein (SASBDB IDs SASDAT4 and SASDAU4, respectively; Mertens *et al.*, 2012 ▸). The distributions of a pool of 10 000 randomized conformations, preserving individual domain structure, are shown as broken lines. The distributions of optimized ensembles selected by the genetic algorithm are shown as blue (WT) and red (MUT) bars, respectively. The decreased width of the distribution of selected structures for mutant relative to wild type indicates a reduction in flexibility, and the observed shift to smaller *R*_g_ values provides evidence of structural compaction. The metrics *R*_flex_ and *R*_σ_ are calculated from the distributions as 82% and 1.0 (wild type), and 45% and 0.1 (mutant). The *R*_flex_ value of the random pool is calculated as 85%.

*EOM* (ensemble optimization method). This method has been widely used to study the potential flexibility of a variety of biological systems (Bernadó *et al.*, 2007 ▸; Tria *et al.*, 2015 ▸). Contrary to *OLIGOMER*, in *EOM* neither the number of components nor their respective scattering contributions are known. Instead, an indirect strategy is employed to categorize the conformational heterogeneity with options to incorporate high-resolution structures of folded protein domains connected by flexible linkers or bound to DNA. Initially a large pool of randomized components, *i.e.* structures, is generated. Then, a genetic algorithm is employed to select a subset of these random components whose average scattering intensity best approximates the experimental SAXS data. In addition, constraints on symmetry, the number of conformers populating the selected ensemble and inter-domain/subunit contacts may be applied. On output, *EOM* provides parameter distributions (*e.g. R*_g_, *D*_max_) for visual assessment of the optimal subset of randomized components of the pool, in terms of compactness and flexibility (Fig. 5 ▸). The metrics *R*_flex_ and *R*_σ_, based on information entropy, have also been introduced (Shannon & Weaver, 1949 ▸; Tria *et al.*, 2015 ▸), to quantitatively characterize the degree of flexibility of the protein. A fully flexible system will provide *R*_flex_ and *R*_σ_ values approaching 1.0, whereas rigid systems yield metrics tending to 0.0.

## 4. Graphical user interfaces   

In addition to scriptable and modular command-line tools, the *ATSAS* package also includes multiple GUIs for manual data manipulation and analysis. Some of the interactive applications available for Windows have not changed significantly since their initial description (Konarev *et al.*, 2003 ▸), and they are not considered here in detail. Instead we shall focus on the new and improved features of the cross-platform data analysis and processing tool *PRIMUS/qt* and the three newcomers to the *ATSAS* package: *POLYSAS*, an interactive graphical system for the analysis of polydisperse systems (Konarev, Volkov & Svergun, 2016 ▸); *SASpy*, a SAS data and model plugin for the cross-platform molecule viewer *PyMOL* (Schroedinger, LLC; http://www.pymol.org); and *CHROMIXS*, a tool for rapid analysis of large SEC-SAXS data sets.

**Figure 6:**
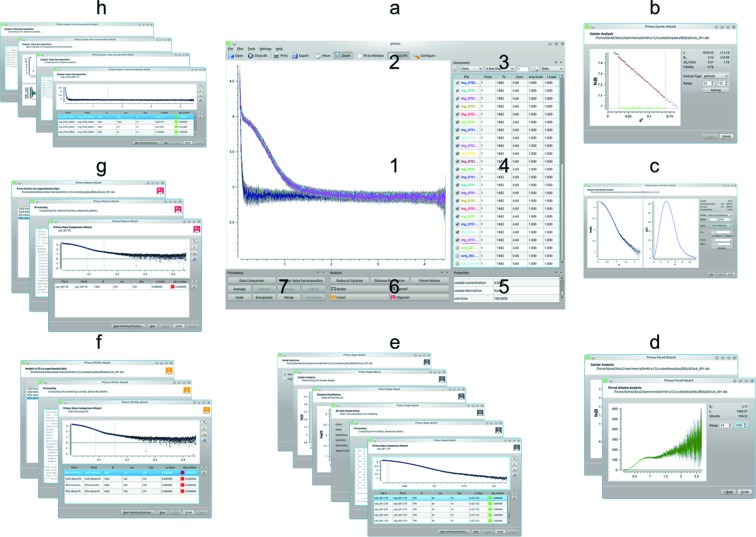
*PRIMUS/qt*, the cross-platform SAS data analysis platform of *ATSAS*, providing (*a*) the main window with (1) a plot area for 1000+ simultaneous datasets, (2) advanced zoom capabilities, (3) advanced file filtering and selection, (4) direct file manipulation, (5) information about the selected file, (6) easily accessible analysis, and (7) data processing options. A variety of analysis wizards are implemented as frontends for convenient and reliable manual analysis of SAS data, employing the various *ATSAS* applications in the background. So far are available (*b*) the Guinier Wizard to determine the radius of gyration, (*c*) the Distance Distribution Wizard to determine the maximum dimension, (*d*) the Porod Wizard to determine the Porod volume, (*e*) the Shape Wizard for *ab initio* shape determination, including averaging and refinement, (*f*) the *CRYSOL* wizard to compute the fit of *a priori* models, (*g*) the *OLIGOMER* wizard for analysis of mixtures with known components, and (*h*) the Singular Value Decomposition Wizard for mixture analysis with unknown components.

*PRIMUS/qt*. The cross-platform implementation of the widely used *PRIMUS* for Windows (Konarev *et al.*, 2003 ▸), named *PRIMUS/qt*, provides all the features of its predecessor and a number of additional functionalities. Data loading, handling, filtering and plotting capabilities make *PRIMUS/qt* a convenient tool for SAS data analysis and interpretation that encompasses basic data reduction and more advanced state-of-the-art analysis, even for complex and large data sets comprising thousands of frames (Graewert & Svergun, 2013 ▸). *PRIMUS/qt* provides convenient access to a variety of the *ATSAS* tools and applications for data processing and analysis (Fig. 6 ▸). These include, but are not limited to, the following: (*b*) the Guinier Wizard to determine *R*_g_ with *AUTORG*/*DATRG*; (*c*) the Distance Distribution Wizard, based on *DATGNOM* and *GNOM*, to determine the regularized scattering fit to the experimental data required for *p*(*r*) profile calculation and *ab initio* modelling; (*d*) the Porod Wizard to manually determine the Porod volume with *DATPOROD*; (*e*) the Shape Wizard for manual or fully automated *ab initio* shape determination with *DAMMIF*, including averaging by *DAMAVER* and refinement with *DAMMIN*; (*f*) the Model Fit wizard to compute the fit from high-resolution models with *CRYSOL* and/or *SREFLEX*; (*g*) the Mixture Wizard for the analysis of mixtures with known components by *OLIGOMER*; and (*h*) the Singular Value Decomposition Wizard to evaluate the number of species in scattering from mixtures with unknown components. In all cases, the wizards guide users through the various analysis procedures in a step-by-step fashion.

**Figure 7:**
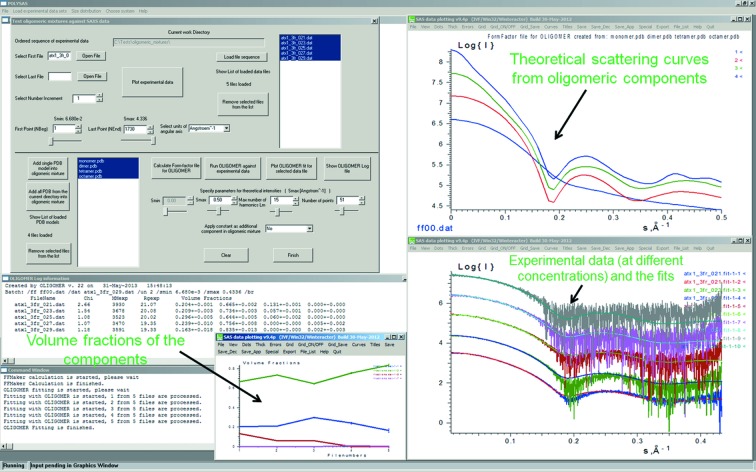
*POLYSAS* GUI for SAXS data modelling of hNGF concentration dependence in solution using an oligomeric mixture of dimers and dimers of dimers (Covaceuszach *et al.*, 2015 ▸).

*POLYSAS* for Windows (Konarev, Volkov & Svergun, 2016 ▸) was developed to simplify the analysis of time-, concentration- or temperature-dependent data collection series (Fig. 7 ▸). Like in *PRIMUS/qt*, the actual data processing is carried out by the previously described tools and applications. *POLYSAS* provides access to an automated processing of the integral structural parameters such as *R*_g_, *D*_max_, MW and *V*_p_ (*via DATTOOLS*) for multiple datasets. It calculates the size distributions for polydisperse systems (*GNOM*) and provides the theoretical intensities from multiple PDB files (*CRYSOL*). It may further be used to estimate the number of components (*SVDPLOT*) and to evaluate the volume fractions of the components for multiple data sets (*OLIGOMER*). Finally, *POLYSAS* provides easy access to quantitative analysis of polydisperse and interactive mixtures with up to five different types of particles (*MIXTURE*). The parameters of the models can be interactively changed using the graphical sliders and the fits are updated automatically *via* the *SASPLOT* graphical viewer (Konarev *et al.*, 2003 ▸).

**Figure 8:**
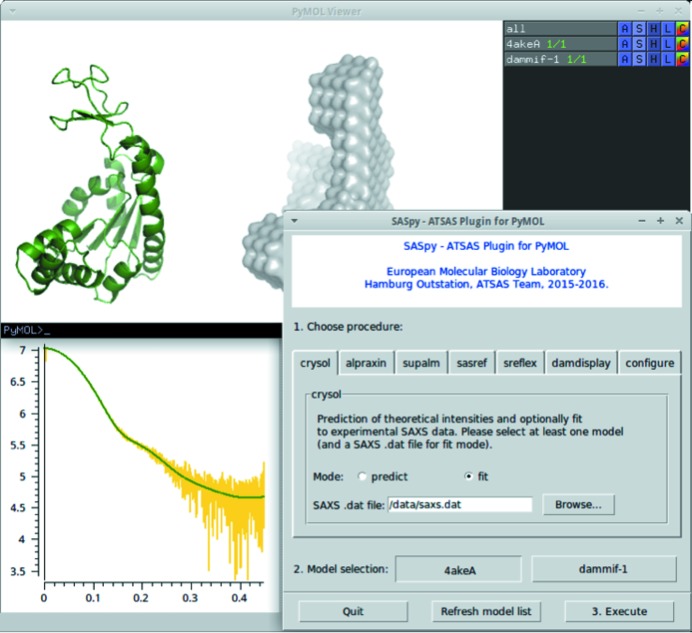
Example of *SASpy* workflow, where structural models can be modified and refined while interactively evaluating their fit to SAXS experimental data.

*SASpy* (Panjkovich & Svergun, 2016*a* ▸) provides a cross-platform graphical interface for the creation and manipulation of hybrid models against SAXS experimental data, similar to the application *MASSHA* (Konarev *et al.*, 2001 ▸). The latter is restricted to the Windows platform and lacks certain features such as mouse-based rearrangement of complexes. *SASpy* is a cross-platform application distributed as an open-source *PyMOL* plugin. Most of *SASpy*’s functionality is provided by *ATSAS* command-line applications, which are executed *via* a few mouse clicks in the plugin window. For example, the user-guided modification of tertiary assemblies is straightforward through the editing features already available in *PyMOL*, while the effects of subunit rearrangements within an assembly on the modelled scattering intensities and subsequent fits to experimental SAXS data can be computed on the fly as *CRYSOL* is executed through the *SASpy* interface (Fig. 8 ▸). *PyMOL* functionality is further expanded by the inclusion of *SUPALM*, which allows superposition of high- and low-resolution models. Automatic refinement tools (*SASREF* and *SREFLEX*) can also be executed through *SASpy*’s point-and-click interface by simply selecting the starting models and the corresponding SAXS data. *SASPy* is open source and available at https://github.com/emblsaxs/saspy.

**Figure 9:**
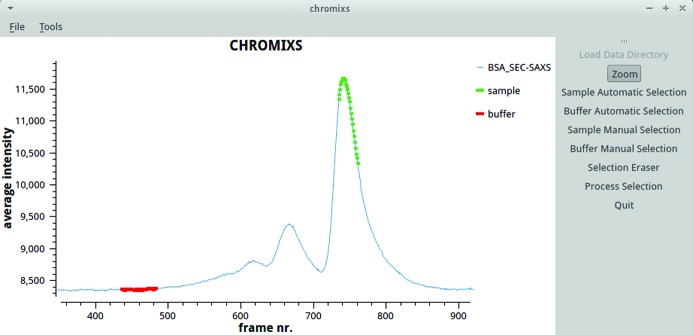
A *CHROMIXS* screenshot displaying the plot (blue) of integrated intensities *versus* time (frame number) for a SEC-SAXS run at the EMBL P12 BioSAXS beamline (Blanchet *et al.*, 2015 ▸). The user has selected a sample region (green) and a buffer region (red) has been predicted automatically by *CHROMIXS*.

*CHROMIXS* (chromatography in-line X-ray scattering). A single SEC-SAXS run may generate thousands of individual SAXS data frames during the online purification process. The GUI-based programs like *PRIMUS/qt* developed to work with a full display of the selected scattering profiles quickly reach their limits when having to handle hundreds or thousands of data files. Within the SEC-SAXS files, the researcher has to identify relevant sections, for example the range of data containing buffer curves and the sample peaks of interest, by a manual, often iterative, selection process. This procedure may require multiple steps that are difficult to formalize and to automate, a fact illustrated by the wide variety of applications developed to aid in the process (Brookes *et al.*, 2016 ▸; Shkumatov & Strelkov, 2015 ▸). *CHROMIXS*, whose previous working title was *SECPLOT*, combines simplicity with automation for processing experimental SEC-SAXS data. It provides an easy to use graphical interface to readily analyse data, allowing calculation of basic structural parameters and *ab initio* models as soon as data collection is completed. Fig. 9 ▸ depicts *CHROMIXS* with a scattering intensity trace obtained from the SEC separation of bovine serum albumin, where suitable buffer- and sample-scattering regions have been selected. The current version includes procedures for basic data reduction and automatic buffer and sample region prediction. For more advanced analysis, such as testing for similarity between individual frames, for example using *DATCMP*, the data are forwarded to *PRIMUS/qt* (manuscript in preparation).

## 5. *ATSAS* online   

Besides the *ATSAS* installation packages for download, academic users may also use the *ATSAS* online web interface which provides access to the cluster facilities at EMBL-Hamburg for longer-running/CPU-heavy modelling applications (https://www.embl-hamburg.de/biosaxs/atsas-online/). About 300 computing cores are available for this service; in 2016, 3300 registered users submitted 46 000 jobs for *DAMMIN*, *DAMMIF*, *CRYSOL*, *GASBOR*, *EOM*, *MONSA* and *SASREF*, and recently, for *CORAL* and other new developments like *AMBIMETER*, *SASRES* and *SREFLEX*. All of the *ATSAS* online web interfaces are designed with ease of use in mind, and hints and links to the application manuals are provided on each page. Upon completion of a submitted job, users receive an email message with a link to the results page, where the job’s output can be visualized and downloaded. Users may access only their own personal projects and data. Additionally, they can check the status of the cluster and their recent submissions at the ‘My Projects’ page, where options are available to report a problem concerning a particular submission to the *ATSAS* online maintainers.

## 6. Conclusions   

The *ATSAS* tools suite provides a comprehensive set of applications for the analysis of small-angle X-ray and neutron scattering data from isotropic solutions of biological macromolecules and nanoparticles. *ATSAS* includes programs for primary data analysis, *ab initio* shape determination and hybrid modelling incorporating structural information from other methods like X-ray crystallography, NMR and EM. For mixtures and polydisperse systems, computations of size distributions and volume fractions of components as well as ensemble analysis are available. The individual command-line tools provide concise interfaces to facilitate their applications, validation or integration into analysis pipelines or graphical user interfaces. The *ATSAS* suite is available for download on all major software platforms (Windows, Mac OS, Linux). Many *ATSAS* programs are available online at the EMBL cluster, and we plan to further extend the online capabilities of the package. *ATSAS* is free for academic users and profits immensely from feedback received from the community. Users are encouraged to post their comments and suggestions at the dedicated branch of the SAXIER forum (http://www.saxier.org/forum).
